# Frontal sinus morphology and maturation determination

**DOI:** 10.1093/ejo/cjag059

**Published:** 2026-08-20

**Authors:** Hatice Kök

**Affiliations:** Faculty of Dentistry, Department of Orthodontics, Selcuk University, Alaeddin Keykubat Campus, Akademi Square, Yeni Istanbul Street No. 309, 42150 Selcuklu, Konya, Türkiye

**Keywords:** frontal sinus, maturity, growth and development, orthodontics, ROC curve

## Abstract

**Aim:**

To determine the relationship between frontal sinus morphometric measurements and established maturation indicators obtained from hand-wrist and lateral cephalometric radiographs, and to evaluate the diagnostic performance of frontal sinus morphometric measurements in discriminating growth and developmental stages.

**Materials and methods:**

This retrospective study analysed radiographs of 240 patients aged 9–19 years who underwent simultaneous hand–wrist and cephalometric imaging. Patients were categorized into six cervical vertebral maturation stages (CVS), with 20 females and 20 males in each stage. Hand–wrist (HWS), cervical vertebra (CVS), and dental maturation (DS) stages were assessed. Frontal sinus measurements included maximum height (FSH_eight_), maximum width (FSW_idth_), frontal sinus index (FSI_ndex_), area (FSA_rea_), and perimeter (FSP_erimeter_). Spearman’s Rho assessed correlations, and ROC analysis evaluated diagnostic performance with cut-off–based accuracy rates. Reliability was examined using the Kappa coefficient, Kendall’s tau-b, and intraclass correlation coefficient (ICC).

**Results:**

After Benjamini–Hochberg false discovery rate (FDR) correction, no significant correlations were found between HWS or DS stages and FSH_eight_ in male patients, or between CVS or DS stages and FSI_ndex_. In females, FSA_rea_ showed the highest discriminatory performance for classifying the transition from pre-pubertal to pubertal stage (AUC = 0.794; *P* < 0.001). In males, FSW_idth_ showed the strongest discriminatory performance (AUC = 0.817; *P* < 0.001). For the transition from the pubertal to post-pubertal stage, FSW_idth_ showed the highest discriminatory performance (AUC = 0.846; *P* < 0.001), and all frontal sinus measurements were statistically significant in both sexes.

**Conclusion:**

Frontal sinus morphometry showed high reliability and meaningful associations with age and maturation indicators. However, as AUC values ranged from moderate to good (0.655–0.846) and diagnostic performance varied by sex, maturation system, and transition stage, frontal sinus morphometry should be regarded as a supplementary rather than a standalone or definitive indicator of skeletal maturation, pending longitudinal validation.

## Introduction

Accurate assessment of skeletal maturation is fundamental to orthodontic diagnosis and treatment planning. Growth-related treatments are most effective during the pubertal growth spurt, a critical transition from childhood to adulthood [1]. The potential for skeletal modification is limited after puberty, and orthodontic treatment may require surgical intervention [2–5]. Skeletal maturation is traditionally assessed via chronological age, anthropometric changes, secondary sexual characteristics, and dental/bone calcification [6]. In orthodontics, radiographic methods are preferred for their integration into routine diagnostic workflows and their ability to monitor longitudinal dental and osseous changes. Noninvasive radiographic assessment has a drawback: radiation exposure [7]. Optimizing the balance between diagnostic yield and radiation dose (ALARA principle) when evaluating growth is essential [8].

The frontal sinuses are bilateral, irregular pneumatic cavities within the frontal bone. Absent at birth, they originate from the migration of anterior ethmoid cells during the first postnatal year, with vertical and lateral expansion toward the orbital roof beginning in the second year. Radiographic visibility is typically achieved by age eight [9–11]. Due to this developmental trajectory, frontal sinus pneumatisation is utilized as a skeletal marker to assess somatic growth and mandibular development [12–14].

Previous studies have investigated the relationship between frontal sinus development and various maturation parameters. Ruf and Pancherz [14–16] demonstrated that frontal sinus development follows a pubertal growth pattern closely paralleling body height velocity and reported that skeletal maturity could be estimated with relatively high accuracy; however, their analyses did not include formal diagnostic modelling or sex-specific evaluation. Rossouw *et al*. [12] reported a correlation between frontal sinus area and mandibular growth indicators, although no standardized maturation staging system was applied. Gagliardi *et al*. [13] extended these findings to include female subjects (*n* = 31) and confirmed cross-cultural validity in an Aboriginal Australian population. Mahmood *et al*. [17] employed a cross-sectional design with CVS staging in a balanced sex sample. However, they evaluated only the frontal sinus index and did not apply ROC-based diagnostic modelling.

Accordingly, several methodological gaps remain in the literature. To our knowledge, previous studies have not comprehensively: (i) applied ROC analysis to determine sex-specific diagnostic cut-off values for pubertal stage discrimination; (ii) simultaneously evaluated three maturation indicators (CVS, hand–wrist, and dental staging) within the same cohort; (iii) assessed multiple frontal sinus measurements, including area and perimeter; (iv) employed a fully balanced sex-stratified design across all CVS; (v) performed measurements using high-precision digital methods with standardized magnification calibration; or (vi) explored the potential of extracting multiple maturation-related measurements from a single routinely acquired lateral cephalometric radiograph in accordance with the ALARA principle. The present study was designed to address these gaps.

The present study, therefore, aimed to determine the relationship between frontal sinus morphometric measurements and established maturation indicators obtained from hand-wrist and lateral cephalometric radiographs and to evaluate the diagnostic performance of frontal sinus morphometric measurements in discriminating growth and developmental stages.

## Materials and methods

**Figure 1:**
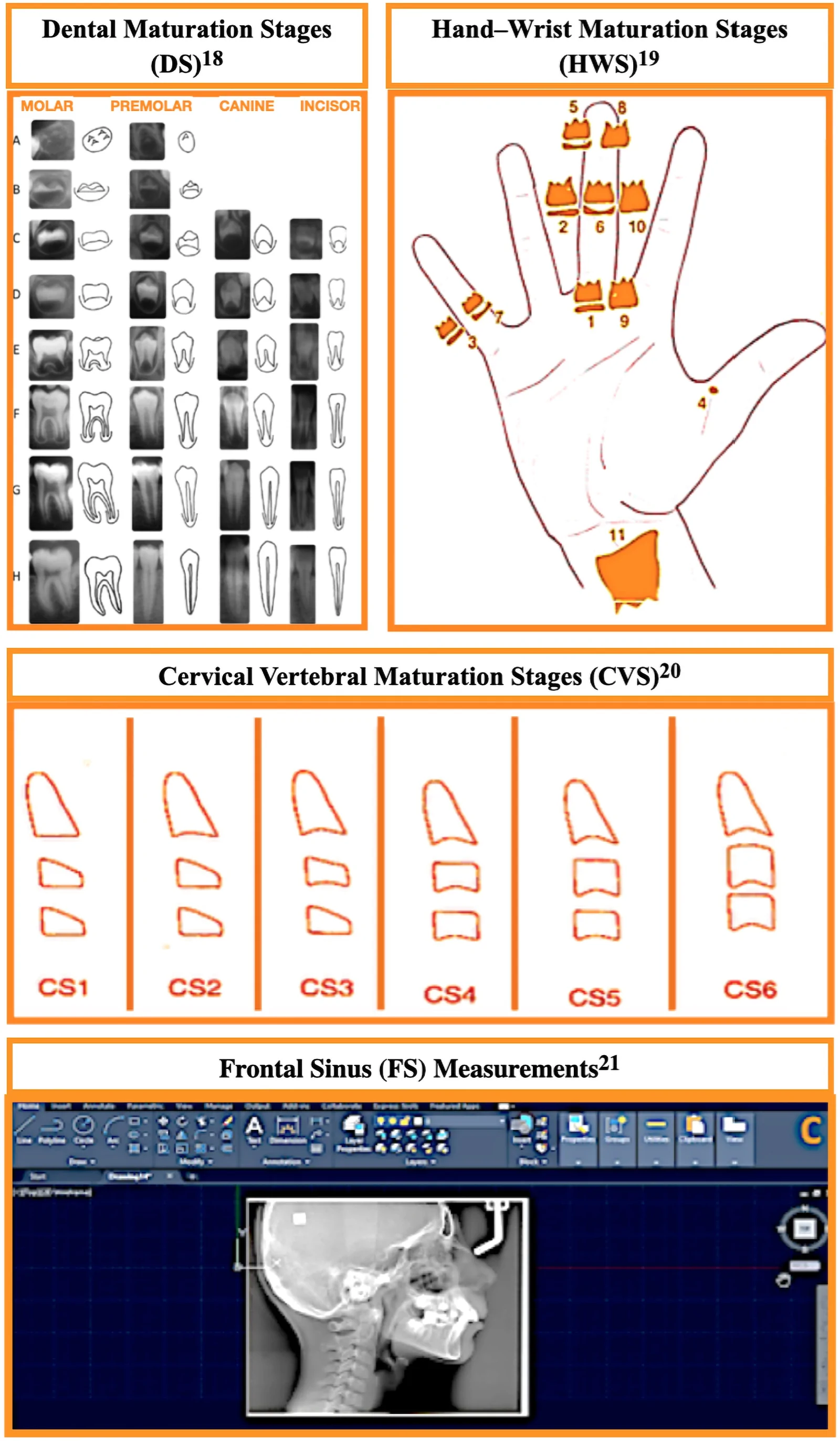
The study measurements and references.

Our retrospective cross-sectional observational study included 240 patients aged 9–19 years who underwent simultaneous hand-wrist and cephalometric radiography at the Department of Orthodontics, Selcuk University Faculty of Dentistry. The cephalometric radiographs were classified into six cervical vertebral maturation stages (CVS), with 20 females and 20 males in each stage. The exclusion criteria included: poor-quality or incomplete radiographic records (asynchronous lateral cephalometric and hand-wrist radiographs); obscured permanent second molars; missing demographic data; history of craniofacial anomalies, syndromes, or systemic diseases affecting growth; and previous orthodontic, orthognathic, or surgical interventions in the head and neck region. After application of the inclusion and exclusion criteria, 240 eligible subjects were included in the final analysis. Because subjects with incomplete radiographic or demographic records were excluded during screening, no missing data were present in the final dataset. The ethics committee approval for the research was obtained in accordance with the decision of the Selcuk University Non-Interventional Clinical Research Ethics Committee dated 24 February 2022 and numbered 2022/11. The morphometric analysis procedure used in this study [18–21] is described in Fig. 1. The maximum frontal sinus height (FSH_eight_) and width (FSW_idth_) were measured on cephalometric radiographs using AutoCAD 2022 (Autodesk Inc., San Rafael, CA, USA), following the Ertürk method [21].

Additionally, the ratio of FSH to width (Frontal Sinus Index, FSI_ndex_), frontal sinus area (FSA_rea_), and frontal sinus perimeter (FSP_erimeter_) were also measured (Fig. 1). To ensure independence between the grouping variable and the outcome measurements, all frontal sinus measurements were performed by a single calibrated examiner who was blinded to the subjects’ age, sex, and maturation stage classifications (CVS, HWS, and DS) at the time of measurement. CVS staging and frontal sinus measurements were conducted in separate sessions. For intra-examiner reliability assessment, 30 radiographs were randomly selected and re-evaluated by the same examiner after a two-week interval to minimize recall bias.

Given the retrospective and cross-sectional design of this study, all associations between frontal sinus morphometric measurements and maturation stages reflect concurrent relationships observed at a single time point and should not be interpreted as predictive or causal. The diagnostic performance metrics derived from ROC analyses indicate that frontal sinus measurements discriminate between maturation-stage classifications within this cross-sectional sample, rather than predict future developmental transitions.

### Statistical analysis

Data were analysed using SPSS 20.0 (IBM Corp., Armonk, NY). Data are presented as mean ± SD (median; Q1–Q3) or frequency (%). Normality was verified via the Kolmogorov–Smirnov test. Group differences were assessed using Mann–Whitney U and Kruskal–Wallis tests (with *post hoc* pairwise comparisons). Correlations were evaluated using Spearman’s rho, and diagnostic performance was evaluated using ROC analysis. Optimal cut-off values for each frontal sinus measurement were determined using the Youden Index (J = Sensitivity + Specificity − 1), which identifies the cut-off value that maximizes the combined sensitivity and specificity. Sensitivity and specificity were calculated for each optimal cut-off and are presented as point estimates with corresponding 95% confidence intervals, calculated using the exact (Clopper–Pearson) binomial method based on the true positive, false negative, true negative, and false positive counts at each cut-off. Positive predictive value, negative predictive value, and accuracy are presented as point estimates, calculated directly from the same counts of true positives, false negatives, true negatives, and false positives. Reliability was confirmed using Kappa, Kendall’s tau-b, and ICC. Significance was set at *P* < 0.05. *P*-values obtained from correlation analyses were adjusted using the Benjamini–Hochberg false discovery rate (FDR) procedure to reduce the risk of false-positive findings due to multiple testing. Partial correlation analyses controlling for chronological age were additionally performed separately for each sex to evaluate age-independent associations between frontal sinus measurements and maturation indicators. No *a priori* sample size calculation was performed; instead, the study employed a balanced stratified sampling design to ensure equal sex representation across all CVS stages. A *post hoc* power analysis conducted using the Hanley and McNeil method—based on the most conservative AUC value observed (AUC = 0.655, male FSH_eight_, pubertal to post-pubertal transition; *n* = 79 post-pubertal, *n* = 84 pubertal; *α* = 0.05) yielded an estimated statistical power of 95.2%, substantially exceeding the conventional 80% threshold. The sample size of 40 subjects per CVS stage is also consistent with the most comparable prior study, Mahmood *et al*. [17] (42 subjects per CVS stage).

## Results

FSA_rea_, FSP_erimeter_, FSW_idth_, and FSH_eight_ demonstrated significant CVS-specific variations in both sexes (*P* < 0.001). FSI_ndex_ varied significantly across CVS in females (*P* = 0.001) but not in males (*P* = 0.481). Additionally, FSA_rea_ (*P* = 0.002), FSP_erimeter_ (*P* = 0.021), and FSW_idth_ (*P* < 0.001) were significantly lower in males than in females. The FSH_eight_ showed no significant sexual dimorphism (*P* = 0.081), whereas the FSI_ndex_ differed significantly between sexes (*P* < 0.001). Significant variations were observed in FSA_rea_, FSP_erimeter_, FSW_idth_, and FSH_eight_ across HWS stages in both sexes (*P* < 0.001). FSA_rea_ and FSP_erimeter_ generally increased across HWS in both sexes. An increase in FSA_rea_ in both sexes and FSP_erimeter_ in males was observed at HWS 3, followed by a decrease at HWS 4. Significant variation in FS measurements was observed according to DS in both sexes (*P* < 0.001), though the FSI_ndex_ remained nonsignificant in males. In males, all FS dimensions increased progressively across subsequent stages. In females, the FSA_rea_, FSP_erimeter_, FSW_idth_, and FSH_eight_ were higher at stage 4, followed by a slight decrease at stage 5, then continued to rise in the later stages.

There was a positive and highly significant correlation between CVS and HWS (*r* = 0.901; *P* < 0.001) and DS (*r* = 0.674; *P* < 0.001) stages in female patients. Chronological age showed significant positive correlations with all maturation stages and FS measurements (*r* = 0.817, *P* < 0.001), but a moderate negative correlation with FSI_ndex_ (*r* = −0.380, *P* = 0.001). The FSA_rea_, FSP_erimeter,_ and FSW_idth_ were consistently and positively associated with HWS in both sexes, whereas the association between FSH_eight_ and HWS did not remain significant after FDR correction. DS stages showed significantly lower correlation coefficients than CVS and HWS. Male patients exhibited similarly high significant positive correlations between all maturation stages (CVS–HWS: *r* = 0.853; CVS–DS: *r* = 0.570) and FS measurements, though coefficients were generally lower than in females. After FDR correction, no significant correlations were found between HWS or DS and FSH_eight_ in males, or between CVS or DS and FSI_ndex_ (Table 1). Partial correlation analyses controlling for chronological age demonstrated that several significant associations between frontal sinus dimensions and maturation indicators persisted independently of age in both sexes. In particular, FSA_rea_, FSP_erimeter,_ FSW_idth_, and FSH_eight_ remained significantly associated with CVS after adjustment for age.

**Table 1:** Correlation values between maturation stages and FS measurements.

Female Rho	FSA_rea_	FSP_erimeter_	FSW_idth_	FSH_eight_	FSI_ndex_	Age
**CVS**	0580**	0519**	0659**	0434**	−0380**	0817**
**HWS**	0542**	0466**	0596**	0415**	−0334*	0739**
**DS**	0473**	0320*	0522**	0244*	−0356**	0575**
**Male Rho**						
**CVS**	0552**	0456**	0566**	0277*	−0165	0799**
**HWS**	0472**	0419**	0516**	0216	−0227	0702**
**DS**	0394**	0320*	0390**	0182	−0152	0535**

Note: Statistical significance was determined after Benjamini–Hochberg FDR correction.

** *P* < 0.01, * *P* < 0.05.

**Figure 2:**
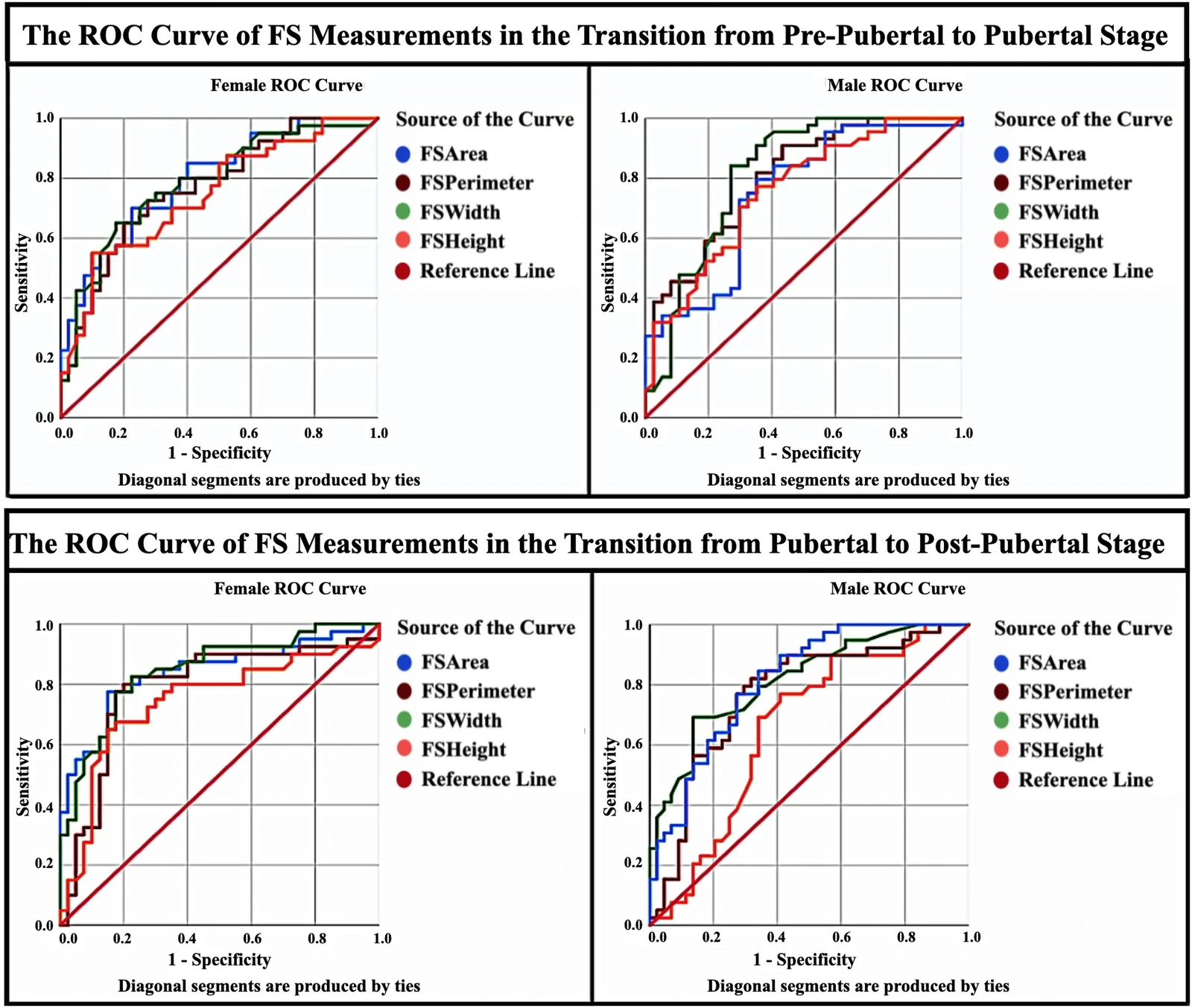
ROC curves of FS measurements in the transition from the pre-pubertal to pubertal stage and from the pubertal to post-pubertal stage.

Using cervical maturation stages, pubertal developmental stages were classified into three stages: pre-pubertal (32.1%), pubertal (35%), and post-pubertal (32.9%). The cut-off and diagnostic rate information for FS measurements at stage transitions were calculated separately for females and males (Tables 2 and 3). Because FSI_ndex_ values did not yield significant results in ROC analysis, diagnostic rates were not calculated. In ROC analyses for the pre-pubertal to pubertal transition, FSA_rea_ demonstrated the highest discriminatory performance in females (AUC = 0.794; *P* < 0.001), whilst FSW_idth_ showed the strongest discriminatory performance in males (AUC = 0.817; *P* < 0.001) (Fig. 2). For the pubertal to post-pubertal transition, FSW_idth_ demonstrated the highest discriminatory performance in females (AUC = 0.846; *P* < 0.001), and all frontal sinus measurements reached statistical significance in both sexes (Fig. 2).

**Table 2:** Diagnostic performance of frontal sinus measurements for the transition from pre-pubertal to pubertal stage.

Measurement	Sex	AUC	AUC 95% CI	*P*-value	Cut-off	Sensitivity (%) 95% CI	Specificity (%) 95% CI	PPV (%)	NPV (%)	Accuracy (%)
**FSA_rea_**	Female	0.794	0.698–0.890	<0.001	345 328.10	70.0 (53.5–83.4)	77.5 (61.5–89.2)	75.7	72.1	73.8
	Male	0.748	0.639–0.856	<0.001	231 940.38	79.5 (64.7–90.2)	64.9 (47.5–79.8)	72.9	72.7	72.8
**FSP_erimeter_**	Female	0.770	0.668–0.872	<0.001	2751.48	72.5 (56.1–85.4)	72.5 (56.1–85.4)	72.5	72.5	72.5
	Male	0.799	0.703–0.895	<0.001	2240.18	90.9 (78.3–97.5)	56.8 (39.5–72.9)	71.4	84.0	75.3
**FSW_idth_**	Female	0.783	0.683–0.884	<0.001	625.11	65.0 (48.3–79.4)	82.5 (67.2–92.7)	78.8	70.2	73.8
	Male	0.817	0.719–0.915	<0.001	511.45	84.1 (69.9–93.4)	73.0 (55.9–86.2)	78.7	79.4	79.0
**FSH_eight_**	Female	0.746	0.639–0.853	<0.001	1172.30	55.0 (38.5–70.7)	90.0 (76.3–97.2)	84.6	66.7	72.5
	Male	0.758	0.654–0.862	<0.001	956.63	77.3 (62.2–88.5)	64.9 (47.5–79.8)	72.3	70.6	71.6

Note: AUC, area under the receiver operating characteristic curve; CI, confidence interval; NPV, negative predictive value; PPV, positive predictive value. Optimal cut-off values were derived using the Youden Index. Sensitivity and specificity are presented as point estimates with corresponding 95% confidence intervals calculated using the exact (Clopper–Pearson) binomial method based on the true positive, false negative, true negative, and false positive counts at each cut-off. PPV, NPV, and accuracy are presented as point estimates.

**Table 3:** Diagnostic performance of frontal sinus measurements for the transition from pubertal to post-pubertal stage.

Measurement	Sex	AUC	AUC 95% CI	*P*-value	Cut-off	Sensitivity (%) 95% CI	Specificity (%) 95% CI	PPV (%)	NPV (%)	Accuracy (%)
**FSA_rea_**	Female	0.839	0.749–0.929	<0.001	546 731.48	75.0 (58.8–87.3)	85.0 (70.2–94.3)	83.3	77.3	80.0
	Male	0.815	0.725–0.904	<0.001	371 703.61	84.6 (69.5–94.1)	65.9 (50.1–79.5)	68.8	82.9	74.7
**FSP_erimeter_**	Female	0.788	0.680–0.896	<0.001	3377.94	82.5 (67.2–92.7)	77.5 (61.5–89.2)	78.6	81.6	80.0
	Male	0.770	0.665–0.875	<0.001	3178.67	82.1 (66.5–92.5)	68.2 (52.4–81.4)	69.6	81.1	74.7
**FSW_idth_**	Female	0.846	0.761–0.931	<0.001	725.24	82.5 (67.2–92.7)	77.5 (61.5–89.2)	78.6	81.6	80.0
	Male	0.816	0.726–0.906	<0.001	635.94	69.2 (52.4–83.0)	86.4 (72.6–94.8)	81.8	76.0	78.3
**FSH_eight_**	Female	0.743	0.628–0.857	0.015	1380.91	67.5 (50.9–81.4)	82.5 (67.2–92.7)	79.4	71.7	75.0
	Male	0.655	0.536–0.775	<0.001	1086.35	89.7 (75.8–97.1)	43.2 (28.3–59.0)	58.3	82.6	65.1

Note: AUC, area under the receiver operating characteristic curve; CI, confidence interval; NPV, negative predictive value; PPV, positive predictive value. Optimal cut-off values were derived using the Youden Index. Sensitivity and specificity are presented as point estimates with corresponding 95% confidence intervals calculated using the exact (Clopper–Pearson) binomial method based on the true positive, false negative, true negative, and false positive counts at each cut-off. PPV, NPV, and accuracy are presented as point estimates.

Intra-examiner reliability was assessed by re-evaluating 30 randomly selected radiographs. All measurements showed high and significant agreement (Table 4).

**Table 4:** Maturation assessments concordance values.

1st and 2nd measurements	Agreement statistic	Agreement value^a^
**CVS**	Kappa	0839
**CVS**	Kendall’s Tau-b	0916
**HWS**	Kendall’s Tau-b	0922
**DS**	Kendall’s Tau-b	0905
**FSA_rea_**	ICC	0998
**FSP_erimeter_**	ICC	0996
**FSW_idth_**	ICC	0997
**FSH_eight_**	ICC	0995
**FSI_ndex_**	ICC	0991

^a^All concordance values are significant at the *P* < 0.001 level.

To evaluate the independence of the observed discriminatory performance from the CVS-based stratification strategy, supplementary ROC analyses were conducted using HWS and DS stages as entirely independent reference standards. Across both independent systems, frontal sinus measurements demonstrated consistent and significant discriminatory performance, with AUC values comparable to or higher than those obtained using CVS as the reference standard. The full results of these supplementary analyses, including AUC values and 95% confidence intervals for all measurements across both reference standards and both pubertal transitions, are presented in Table 5.

**Table 5:** Independent corroboration of frontal sinus diagnostic performance: ROC analyses against HWS and DS maturation stages.

Measurement	HWS pre→Pub aUC (95% CI)	*P* value	HWS pub→Post aUC (95% CI)	*P* value	DS pre→Pub aUC (95% CI)	*P* value	DS pub→Post aUC (95% CI)	*P* value
**FSA_rea_**	0.596 (0.492–0.701)	0.071	0.825 (0.767–0.882)**	<0.001	0.827 (0.759–0.895)**	<0.001	0.820 (0.765–0.875)**	<0.001
**FSP_erimeter_**	0.693 (0.598–0.788)**	<0.001	0.778 (0.713–0.844)**	<0.001	0.833 (0.766–0.901)**	<0.001	0.776 (0.713–0.839)**	<0.001
**FSW_idth_**	0.609 (0.505–0.714)*	0.040	0.813 (0.754–0.872)**	<0.001	0.795 (0.718–0.871)**	<0.001	0.839 (0.787–0.892)**	<0.001
**FSH_eight_**	0.692 (0.597–0.787)**	<0.001	0.716 (0.645–0.788)**	<0.001	0.819 (0.747–0.890)**	<0.001	0.711 (0.642–0.780)**	<0.001
**FSI_ndex_**	0.558 (0.453–0.663)	0.276	0.339 (0.261–0.416)**	<0.001^a^	0.524 (0.412–0.636)	0.674	0.291 (0.222–0.359)**	<0.001^a^

Note: AUC, area under the curve; CI, confidence interval; DS, dental maturation stages; HWS, hand-wrist skeletal maturation stages; Pre→Pub, pre-pubertal to pubertal transition; Pub→Post, pubertal to post-pubertal transition.

*P < 0.05; **P < 0.001; no annotation: *P* > 0.05.

^a^FSI_ndex_ values below 0.5 indicate an inverse association, consistent with the known decrease in the FS height-to-width ratio with advancing skeletal maturation.

## Discussion

The present study builds on a well-established body of literature linking frontal sinus development to somatic and skeletal maturation. Ruf and Pancherz [14–16] established the frontal sinus as a marker of skeletal maturation in males, a finding that remains the primary reference point for the field. Rossouw *et al*. [12] identified frontal sinus (FS) morphometry as an auxiliary indicator of growth. Gagliardi *et al*. [13] confirmed a well-defined pubertal spurt in frontal sinus growth across sexes and ethnic populations, and Mahmood *et al*. [17] established, in a balanced cross-sectional sample (*n* = 252), that FS height and width increase significantly across CVS stages, whilst the FS index alone is insufficient for pubertal stage discrimination. The present study sought to address the methodological gaps inherent to these designs: the restriction to male subjects in longitudinal work, the absence of ROC-based diagnostic modelling, the lack of simultaneous multi-indicator maturation assessment, and the limited range of frontal sinus measurements evaluated.

Against this background, the present study offers six methodological advances over prior work. First, ROC-based threshold analysis was applied to evaluate the diagnostic performance of frontal sinus morphometry, yielding sex-specific cut-off values, along with sensitivity, specificity, and accuracy metrics for each measurement, thereby directly addressing the gap identified by Ruf and Pancherz [16], who explicitly called for future research in female populations. Second, three concurrent maturation indicators (CVS, HWS, and DS) were evaluated simultaneously within the same cohort, enabling direct comparison of their relationships with frontal sinus dimensions; no previous study had assessed all three together. Third, five frontal sinus measurements (FSH_eight,_ FSW_idth_, FSA_rea_, FSP_erimeter_, and FSI_ndex_) were assessed, including area and perimeter, which have been largely unexplored in maturation research; notably, FSA_rea_ demonstrated the strongest discriminatory performance for pubertal onset detection in females (AUC = 0.794). Fourth, a fully balanced sex-stratified design was employed across all six CVS stages (20 females and 20 males per stage; total *n* = 240), enabling robust sex-specific analyses. Fifth, measurements were performed using AutoCAD 2022, with radiographic magnification standardized against a known reference scale, thereby minimizing measurement error compared with earlier manual tracing or digitiser-based approaches. Sixth, consistent with the ALARA principle, this study demonstrates that maximum developmental information (encompassing frontal sinus morphometry, cervical vertebral maturation, and dental maturation) can be extracted from a single routinely acquired lateral cephalometric radiograph, potentially reducing the need for additional radiation exposure from supplementary hand-wrist radiographs.

Whilst frontal sinus expansion from childhood to adulthood is well documented, its precise aetiology remains multifactorial, encompassing mucosal growth and resorption dynamics [22], bone density and trabecular remodelling [23], intracranial pressure gradients [24], and genetic and morphological variation [25]. Based on the radiographic visibility of the frontal sinus from age eight [26], patients aged nine and older were included, aligning with typical ages for orthodontic consultations. Although inclusion of the earliest CVS stage was sufficient for the study objectives, evaluating younger cohorts could further elucidate early frontal sinus ontogeny.

Valverde *et al*. [27] reported frontal sinus widths ranging from 10.78 to 13.56 mm in Japanese females aged ≥8 years. In our cohort, the lowest mean FSW_idth_ was 10.97 ± 2.99 mm in males and 13.35 ± 2.19 mm in females, suggesting relatively advanced sinus development despite early skeletal maturation. Significant increases in FSW_idth_ were observed between initial and advanced stages, with females exhibiting significantly higher values than males (*P* < 0.001). Contrary to reports by Hanson and Owsley [28] and Ponde *et al*. [29], who observed male predominance in frontal sinus dimensions in Eskimo and Brazilian samples, respectively, the present study found FSA_rea_ (*P* = 0.002), FSP_erimeter_ (*P* = 0.021), and FSW_idth_ (*P* < 0.001) to be significantly lower in males than in females, whilst FSH_eight_ showed no significant sexual dimorphism (*P* = 0.081) and FSI_ndex_ differed significantly between sexes (*P* < 0.001). Several potential explanations merit consideration. First, the observed pattern may reflect population-specific sexual dimorphism, as frontal sinus morphology is known to vary considerably across ethnic groups and geographic populations [17, 28, 29]. Second, since females reach equivalent CVS at younger chronological ages than males, they may have had more cumulative time for frontal sinus pneumatisation to proceed at each stage, potentially resulting in relatively larger dimensions at equivalent skeletal maturation stages. Third, confounding by age distribution within CVS cannot be excluded; if females within each stage were systematically older than their male counterparts, this could contribute to larger sinus dimensions independently of biological sex differences. Fourth, differences in stage composition between sexes may also have influenced the observed dimensional differences. These explanations remain speculative in the absence of longitudinal data and age-matched comparisons within stages, and the sex difference observed in this study may be population-specific and not generalizable to other ethnic groups. Future studies with age-matched sex comparisons within each maturation stage would be necessary to disentangle the relative contributions of biological, developmental, and methodological factors to this finding.

Brown *et al*. [30] suggested that frontal sinus enlargement concludes by age 14 in girls and age 16 in boys, noting its unreliability for detecting the pubertal peak. Harris *et al*. [31] reported that the frontal sinus becomes anatomically detectable at approximately 1 year of age, with growth continuing into the post-pubertal period and reaching full maturity by approximately age 20. Ruf and Pancherz [15] demonstrated that frontal sinus expansion in males peaks 1.4 years after peak height velocity, with skeletal maturity predictable within 1- and 2-year intervals at 85% and 75% certainty, respectively. Ruf and Pancherz [16] reported high accuracy in identifying post-peak growth (96.3% and 88.5% for 1- and 2-year predictions), but lower reliability for timing peak height velocity (55.5% and 57.7%, respectively). Overall, FS measurements demonstrated significant cross-sectional discriminatory performance across both pubertal transitions (Tables 2 and 3). Valverde *et al*. [27] noted that frontal sinus enlargement in Japanese girls correlated with increases in height during the pubertal spurt. Due to its retrospective design, the present study lacked data on patient height and mandibular growth, which may be considered a limitation. To evaluate the consistency of the findings across independent maturation systems, supplementary ROC analyses were conducted using HWS and DS stages as entirely independent reference standards (Table 5). Across both independent reference standards, frontal sinus measurements demonstrated consistent and significant discriminatory performance, with AUC values comparable to or exceeding those obtained using CVS as the reference standard. The sole exception was FSA_rea_, for the HWS-based pre-pubertal to pubertal transition (AUC = 0.596, *P* = 0.071), which may reflect the inherent lower sensitivity of HWS staging for early pubertal detection compared with CVS and DS. All remaining measurements and all pubertal to post-pubertal analyses were significant across both independent reference standards, confirming the robustness of the observed associations.

Consistent with the present findings, Gagliardi *et al*. [13] reported a close relationship between the frontal sinus and hand-wrist maturation in both sexes. In contrast, Patil and Revankar [32] reported no significant correlation between the FS index and the MP3 maturation stage. Similarly, FSI_ndex_ demonstrated limited discriminatory performance in the present study, supporting the view that ratio-based measurements may be less reliable maturation indicators than absolute dimensional measures. In male patients, no significant correlation was observed between FSH_eight_ and HWS stages, and no significant differences were found in FSI_ndex_ across CVS or DS stages, although significant differences were detected across HWS stages. All frontal sinus measurements correlated positively and highly significantly with HWS, with somewhat lower but still significant coefficients for DS than for CVS and HWS. All frontal sinus measurements (except FSI_ndex_) were also significantly and positively intercorrelated. All maturation stage classifications and frontal sinus measurements were significantly correlated with chronological age. Positive and highly significant correlations were observed between all maturation stages (CVS-HWS: *r* = 0.853; CVS-DS: *r* = 0.570) and frontal sinus measurements in male patients, with correlation coefficients slightly lower than those observed in females. After FDR correction, no significant correlations remained between HWS or DS stages and FSH_eight_ in males, or between CVS or DS stages and FSI_ndex_. The stronger correlation of frontal sinus dimensions with skeletal maturation (CVS and HWS) than with dental maturation (DS) likely reflects shared somatotrophic growth patterns between the craniofacial complex and the general skeleton, whereas dental development is often influenced more by genetic and localized factors. Mahmood *et al*. [17] observed greater FS height and width in males but a higher ratio in females, reporting a low negative correlation between CVS and frontal sinus dimensions in males (tau-b = −0.271; *P* < 0.001) but no significant correlation in females (tau-b = −0.006; *P* = 0.928). In contrast, the present study found positive and significant correlations between all frontal sinus measurements and CVS in both sexes (except for FSI_ndex_ in males). The observed AUC values ranged from moderate to good (0.655–0.846). The lowest accuracy was recorded for male FSH_eight_ during the pubertal to post-pubertal transition (AUC = 0.655, accuracy 65.1%), indicating that not all measurements perform equally across subgroups and suggesting more limited utility in borderline clinical assessment. FSI_ndex_ did not yield significant ROC results in either sex, and FSH_eight_ showed no significant correlation with HWS or DS stages in males, highlighting inconsistencies in certain measurements. These findings support the use of frontal sinus morphometry as a complementary tool that supports, but does not replace, CVS staging in specific clinical situations. Since frontal sinus measurements are obtained from the same lateral cephalometric radiograph already used for CVS staging, no additional radiation exposure is required. In this context, frontal sinus morphometry may warrant further investigation as an adjunctive morphometric parameter in clinically equivocal cases (e.g., when CVS staging yields ambiguous results between stages 2–3 or stages 4–5), where treatment timing decisions are most critical. In such cases, FSA_rea_ and FSW_idth_ generally demonstrated the strongest discriminatory performance across the evaluated transition stages. For the pre-pubertal to pubertal transition, FSA_rea_ achieved an AUC of 0.794 in females, and FSW_idth_ achieved an AUC of 0.817 in males, with accuracies of 73.8% and 79%, respectively. For the pubertal to post-pubertal transition, FSW_idth_ achieved the highest AUC of 0.846 in females (accuracy 80.0%), and FSA_rea_ achieved an AUC of 0.815 in males (accuracy 74.7%). Although these performance levels are insufficient for independent clinical decision-making, they suggest that FSA_rea_ and FSW_idth_ may provide useful additional information when CVS staging alone is inconclusive. However, the incremental diagnostic benefit of frontal sinus morphometry over CVS staging alone has not been formally quantified in the present study, and future research employing integrated statistical models would be necessary to establish this. Rossouw*et al*. [12] demonstrated significant correlations between frontal sinus size and both maxillary-mandibular length and condylar dimensions in 103 subjects, providing early evidence for the utility of frontal sinus morphometry as an auxiliary growth indicator. The present study extends this work by applying ROC-based diagnostic modelling to quantify the discriminatory performance of individual frontal sinus measurements across pubertal transition stages, an analytical step that was not undertaken by Rossouw *et al*. [12]. Additional cephalometric measurements were not performed here because our study focused on identifying the relationship between frontal sinus dimensions and maturation stages (hand-wrist, dental, and vertebral). Future longitudinal studies using dental tomography with larger samples could address the current limitations of our study. Gupta *et al*. [33] found concordance between dental and hand-wrist maturation in monozygotic twins, but not in dizygotic twins aged 13–15. Although some studies suggested that dental maturation was insufficient for identifying the pubertal spurt [34, 35], Perinetti *et al*. [36] reported that mandibular tooth maturation before the spurt could be informative. Kama *et al*. [37] reported that although mandibular third molar maturation strongly correlated with the pubertal spurt, it was insufficient for its detection. Our study focused on the mandibular second molar in accordance with the ALARA principle; this measure showed the expected correlation with chronological age but did not demonstrate significant associations with FSH_eight_ or FSI_ndex_, consistent with the overall findings of the present study.

### Limitations

A potential methodological concern relates to the use of CVS staging both as the basis for subject stratification and as the primary reference standard in ROC analyses. However, several considerations mitigate this concern: the grouping variable (CVS) and the outcome variable (frontal sinus measurements) are anatomically and methodologically distinct, and all frontal sinus measurements were performed with the examiner blinded to CVS. Furthermore, ROC analyses conducted with HWS and DS as entirely independent reference standards (Table 5) yielded AUC values comparable to or higher than those obtained with CVS, providing direct empirical evidence that the observed discriminatory performance reflects genuine associations rather than artefacts of the stratification strategy. Nevertheless, the balanced design may have marginally affected ROC performance estimates compared with an unselected clinical population, and future studies with independent sampling strategies and external validation cohorts are recommended.

The retrospective and cross-sectional design of the present study represents a fundamental limitation that must be acknowledged. Cross-sectional observations do not permit causal or longitudinal inference; all associations reported here reflect concurrent relationships between frontal sinus dimensions and maturation stage classifications at a single time point. Accordingly, the term ‘discriminatory performance’ has been used throughout in preference to ‘predictive performance,’ and all ROC-derived metrics should be interpreted within this cross-sectional framework. Longitudinal studies with repeated measurements across developmental stages would be necessary to establish true predictive relationships and to validate the diagnostic thresholds identified here.

The absence of an *a priori* sample size calculation represents a methodological limitation. Although the *post hoc* power analysis demonstrated adequate statistical power for all primary ROC analyses, future studies should incorporate prospective power calculations based on expected effect sizes derived from the existing literature.

No internal validation or cross-validation of the ROC-derived diagnostic thresholds was performed. The reported accuracy metrics, sensitivity, specificity, and AUC values are therefore derived from the same dataset used to generate the cut-off values, and may be optimistic relative to performance in an independent external sample. Future studies incorporating prospective validation cohorts are necessary to confirm the clinical applicability of the diagnostic thresholds reported here. In addition, the study population was drawn from a single institution, and population-specific growth patterns may further limit the generalizability of the reported cut-off values to other populations or clinical settings.

Another limitation is the absence of anthropometric growth indicators, such as height velocity or body growth measurements, which may provide additional information regarding pubertal maturation status. Future studies integrating craniofacial, skeletal, and anthropometric developmental measurements may help establish more comprehensive models for maturation assessment.

## Conclusion

Frontal sinus morphometry, specifically the measurement of FSA_rea_ and FSW_idth_, demonstrated meaningful associations with skeletal maturation stages and showed moderate-to-good discriminatory performance for pubertal stage classification. In females, FSA_rea_ showed the strongest discriminatory performance for pubertal onset classification, whilst FSW_idth_ demonstrated the highest diagnostic accuracy for post-pubertal stage discrimination. However, given the moderate-to-good AUC values (0.655–0.846) and the variability in measurement significance and diagnostic performance across sexes, maturation systems, and transition stages, frontal sinus morphometry should be regarded as a supplementary rather than a standalone or definitive indicator of skeletal maturation. All measurements showed high reliability and significant correlations with age and maturation stages, supporting their potential use as supplementary radiographic markers in orthodontic assessment and treatment planning, pending longitudinal validation.

## Data Availability

The datasets generated and/or analysed during the current study are not publicly available due to ethical and privacy restrictions. However, they are available from the corresponding author upon reasonable request, in accordance with the relevant regulations.
